# Disentangling the autism−anxiety overlap: fMRI of reward processing in a community-based longitudinal study

**DOI:** 10.1038/tp.2016.107

**Published:** 2016-06-28

**Authors:** N Mikita, E Simonoff, D S Pine, R Goodman, E Artiges, T Banaschewski, A L Bokde, U Bromberg, C Büchel, A Cattrell, P J Conrod, S Desrivières, H Flor, V Frouin, J Gallinat, H Garavan, A Heinz, B Ittermann, S Jurk, J L Martinot, M L Paillère Martinot, F Nees, D Papadopoulos Orfanos, T Paus, L Poustka, M N Smolka, H Walter, R Whelan, G Schumann, A Stringaris

**Affiliations:** 1 Department of Child and Adolescent Psychiatry, King's CollegeLondon, Institute of Psychiatry, Psychology & Neuroscience, London, UK; 2 NIHR Biomedical Research Centre and Dementia Unit at SouthLondon and Maudsley NHS Foundation Trust and the Institute of Psychiatry, Psychology & Neuroscience, King's College London, London, UK; 3 Section on Development and Affective Neuroscience, National Institute of Mental Health, Bethesda, MD, USA; 4 Institut National de la Santé et de la Recherche Médicale, INSERM Unit 1000 “Neuroimaging & Psychiatry”, Service Hospitalier Frédéric Joliot, Orsay, France; 5 University Paris-Sud 11, Orsay, France; 6 University Paris Descartes - Sorbonne Paris Cité, Paris, France; 7 Psychiatry Department, Orsay Hospital, Orsay, France; 8 Department of Child and Adolescent Psychiatry and Psychotherapy, Central Institute of Mental Health, Medical Faculty Mannheim, Heidelberg University, Mannheim, Germany; 9 Discipline of Psychiatry, School of Medicine and Trinity College Institute of Neuroscience, Trinity College, Dublin, Ireland; 10 University Medical Centre Hamburg-Eppendorf, Hamburg, Germany; 11 Medical Research Council - Social, Genetic and Developmental Psychiatry Centre, Institute of Psychiatry, Psychology & Neuroscience, King's College London, London, UK; 12 Department of Psychiatry, Universite de Montreal, CHU Ste Justine Hospital, Montreal, QC, Canada; 13 Department of Psychological Medicine and Psychiatry, Institute of Psychiatry, Psychology & Neuroscience, King's College London, London, UK; 14 Department of Cognitive and Clinical Neuroscience, Central Institute of Mental Health, Medical Faculty Mannheim, Heidelberg University, Mannheim, Germany; 15 Neurospin, Commissariat à l'Energie Atomique, CEA-Saclay Center, Paris, France; 16 Department of Psychiatry and Psychotherapy, University Medical Center Hamburg-Eppendorf, Hamburg, Germany; 17 Department of Psychiatry, University of Vermont, Burlington, VT, USA; 18 Department of Psychology, University of Vermont, Burlington, VT, USA; 19 Department of Psychiatry and Psychotherapy, Campus Charité Mitte, Charité, Universitätsmedizin Berlin, Berlin, Germany; 20 Physikalisch-Technische Bundesanstalt, Braunschweig, Germany; 21 Department of Psychiatry and Neuroimaging Center, Technische Universität Dresden, Dresden, Germany; 22 AP-HP, Department of Adolescent Psychopathology and Medicine, Maison de Solenn, Cochin Hospital, Paris, France; 23 Rotman Research Institute, Baycrest, Toronto, ON, Canada; 24 Child Mind Institute, New York, NY, USA; 25 Department of Psychology, University of Toronto, Toronto, ON, Canada; 26 Department of Psychiatry, University of Toronto, Toronto, ON, Canada; 27 Department of Child and Adolescent Psychiatry and Psychotherapy, Medical University of Vienna, Vienna, Austria; 28 Department of Psychology, University College Dublin, Dublin, Ireland

## Abstract

Up to 40% of youth with autism spectrum disorder (ASD) also suffer from anxiety, and this comorbidity is linked with significant functional impairment. However, the mechanisms of this overlap are poorly understood. We investigated the interplay between ASD traits and anxiety during reward processing, known to be affected in ASD, in a community sample of 1472 adolescents (mean age=14.4 years) who performed a modified monetary incentive delay task as part of the Imagen project. Blood-oxygen-level dependent (BOLD) responses to reward anticipation and feedback were compared using a 2x2 analysis of variance test (ASD traits: low/high; anxiety symptoms: low/high), controlling for plausible covariates. In addition, we used a longitudinal design to assess whether neural responses during reward processing predicted anxiety at 2-year follow-up. High ASD traits were associated with reduced BOLD responses in dorsal prefrontal regions during reward anticipation and negative feedback. Participants with high anxiety symptoms showed increased lateral prefrontal responses during anticipation, but decreased responses following feedback. Interaction effects revealed that youth with combined ASD traits and anxiety, relative to other youth, showed high right insula activation when anticipating reward, and low right-sided caudate, putamen, medial and lateral prefrontal activations during negative feedback (all clusters *P*_FWE_<0.05). BOLD activation patterns in the right dorsal cingulate and right medial frontal gyrus predicted new-onset anxiety in participants with high but not low ASD traits. Our results reveal both quantitatively enhanced and qualitatively distinct neural correlates underlying the comorbidity between ASD traits and anxiety. Specific neural responses during reward processing may represent a risk factor for developing anxiety in ASD youth.

## Introduction

Anxiety is common in youth with autism spectrum disorder (ASD)^1, 2, 3, 4, 5^ and in young people with sub-diagnostic autistic traits.^6, 7^ Comorbid anxiety causes significant functional impairment in young people with ASD^8, 9^ and impacts on the quality of life of their families.^10^ However, the mechanisms of this association are poorly understood. While considerable research has examined the neural correlates of anxiety in adolescents, few studies have examined these correlates in children with symptoms of ASD. Here we investigate whether aberrations in reward processing underlie the co-occurrence of ASD traits and anxiety and whether they predict the *new* onset of anxiety in youth with ASD traits.

Reward processing has been proposed to be central to ASD,^11, 12^ with aberrant processing of primary,^13^ social^14, 15^ and monetary rewards^16^ reported in children and young people with ASD. Furthermore, studies in youth with ASD have reported associations between brain activations during reward processing and ASD traits such as social communication difficulties^17^ and restricted and repetitive behaviors.^18^ Some^15, 19^ but not all studies^16^ have suggested that the extent to which reward processing in youth with ASD differs from typically developing controls may depend on reward type.

Surprisingly, however, the question whether these reward aberrations are inherent to ASD symptoms or related to disorders that co-occur with ASD remains unanswered. This is a key question considering that less than 10% of children with ASD are free of any concomitant disorders according to some studies.^20, 21^ Anxiety disorders and behavioral difficulties are consistently identified as the most common comorbidities in youth with ASD.^9, 22^ These comorbid disorders are associated with aberrant reward processing in their own right, and therefore could influence reward processing in youth with ASD. Young people with anxiety show disrupted frontostriatal activation when anticipating reward^23, 24, 25^ and when receiving reward feedback.^26^ Youth with behavioral difficulties, in particular oppositional defiant disorder (ODD) symptoms and irritability, often show aberrant responses when rewards fail to appear, perhaps due to the frustrative nature of negative outcomes.^27, 28^

The aim of the present study is to use functional magnetic resonance imaging (fMRI) to disentangle the interplay of ASD traits and anxiety during reward processing. To enable the investigation of these factors, we use a large, community-based sample of adolescents with high vs low levels of ASD traits and anxiety symptoms, who completed a widely used, modified monetary incentive delay (MID) fMRI reward task. Our aim is to distinguish between two important models of comorbidity. One model assumes that neural correlates of comorbidity simply reflect the co-occurrence of neural mechanisms seen with each ASD traits and anxiety separately.^29, 30^ In the other model, the neural correlates of the comorbidity are unique, that is, not seen with any of the two disorders. This distinction is crucial from an etiological and clinical perspective, as finding unique correlates would suggest that the comorbidity might represent a separate nosological process, what has been termed a ‘third independent disorder'.^31^ In addition, our aim is to establish the potential predictive value of neural correlates of comorbidity. Using a longitudinal design, we examine whether neural correlates found in people with concurrent ASD traits−anxiety comorbidity can be used to predict the likelihood of new-onset anxiety in those with high ASD traits only. We achieve this aim in two steps.

First, we test the independent influence of each ASD traits and anxiety on brain correlates of reward processing, and also examine possible interaction effects between ASD traits and anxiety. The latter is particularly important in order to assess whether combined ASD traits and anxiety is associated with distinct etiological mechanisms.^30^

Second, we assess whether the brain activations found in our cross-sectional interaction analyses represent a biomarker that also predicts *successive comorbidity*^32^ between ASD traits and anxiety, that is, whether such brain activations predict the new onset of anxiety in those with high ASD traits. To achieve this, we run regression models with anxiety at 2-year follow-up as the outcome, and brain activations (relevant to the comorbid group) as the predictor of interest, separately for participants with low vs high ASD traits, controlling for baseline anxiety. To capture major elements of reward processing, we enquire about two key stages: reward anticipation and reward feedback. The former is often associated with heightened frontostriatal activation in youth with anxiety symptoms,^23, 24, 25, 33^ whereas negative feedback is likely to elicit frustration, possibly related to irritability/ODD symptoms that are common in ASD^1, 5^ but also in anxiety.^34^ For completeness, we also examine positive reward feedback, since youth with ASD tend to show reduced responsiveness to rewards in fMRI studies.^35, 36^ By using a community sample we avoid the risk of referral bias typical of clinical or convenience samples, a pertinent issue when investigating comorbidity,^29^ although the extent to which our results translate to youth who meet the diagnostic criteria for ASD remains to be tested. We also follow the emerging evidence that ASD traits, but also the mechanisms underlying them, fall on a continuum within the general population.^37, 38^

## Materials and methods

### Participants

Data were obtained from the Imagen database established across eight sites in France, UK, Ireland and Germany, which includes 2223 adolescents recruited in schools. We used data from the first (age around 14 years) and second waves (age around 16 years) of Imagen. Recruitment and assessment procedures were described in detail previously.^39^ All local ethics research committees approved the study. Written informed consent was obtained from a parent or guardian, and verbal assent was obtained from the adolescent. Any adolescents with IQ<70 were excluded from this study. After quality control for neuroimaging and behavioral tests, final sample sizes were 1472 for reward anticipation, 1601 for negative feedback and 1726 for positive feedback. Differences in sample sizes across conditions are due to some fMRI contrasts being non-estimable for some participants at the first-level analysis stage. Calculation of the optimum number of participants needed for an fMRI study is difficult to perform *a priori*. Conventional calculations to compute statistical power for a given effect size are not applicable in imaging, primarily because the MR signal in each voxel has a large degree of spatial and temporal variability. Previous studies assessing the reliability of fMRI group-level analyses have suggested that a sample size of *n=*20−24 is sufficient to obtain good statistical power and accurate activation maps.^40, 41, 42^

### Measures

*IQ* was estimated with the Wechsler Intelligence Scale for Children—Fourth Edition, WISC-IV,^43^ in wave 1 and entered into Psytools (Delosis, London), an online computer platform. Two standardized indices were calculated from the WISC subtests applied during neuropsychological testing for Imagen: Verbal Comprehension (derived from Vocabulary and Similarities subtests) and Perceptual Reasoning (derived from Block Design and Matrix Reasoning subtests).

*ASD traits* were assessed in wave 1 using the ASD section from the Development and Well-Being Assessment (DAWBA;^44^ www.dawba.info), a parent-reported, self-administered structured diagnostic interview with 15 questions about social difficulties, 14 questions about restricted, repetitive behaviors and interests, and three questions about language development to assess DSM-IV-defined ASD symptoms. The diagnostic algorithm derived from the DAWBA ASD module shows strong agreement with that from the Autism Diagnostic Interview—Revised^45, 46^ and has a high predictive value for ASD diagnoses in community settings.^47^ In line with the newest characterization of ASD,^48^ we classified participants as having ‘high' levels of ASD traits if their parents/carers reported three or more symptoms on the social difficulties subscale and three or more symptoms on the restricted and repetitive behaviors subscale of the DAWBA ASD section. Based on this criterion close to 5% of the sample had high ASD traits (Table 1), consistent with the prevalence of clinically relevant autistic traits reported in previous population-based studies.^49^

*Anxiety, ODD and depression* prevalences were estimated based on the established and widely used DAWBA computer algorithm,^50, 51^ which indicates the probability of receiving a DSM-IV-defined diagnosis based on answers provided during the interview. DAWBA algorithm band 2 or above was chosen as a cut-off for relevant psychiatric symptoms. Participants were classified as having ‘any anxiety' if they scored at band 2 or above for at least one DSM-IV anxiety disorder; this algorithm identified 24% of the sample (see Table 1).

*Emotional symptoms* were assessed using the parent-reported emotional symptoms subscale from the Strengths and Difficulties Questionnaire (SDQ).^52^ The scale includes three questions about anxiety, one question about somatic symptoms and one about low mood. A score of 5 and above indicates substantial risk of clinically significant emotional problems^52^ and was used as a cut-off. We used emotional symptoms instead of ‘any anxiety' in confirmatory analyses (see below) to ensure that our findings were not measure-specific.

*Additional relevant symptoms* of hyperactivity, conduct problems and functional impairment were assessed using the parent-reported SDQ.^52^

### Modified MID task

In wave 1, the participants performed a modified version of the well-established MID task^53, 54^ to study neural responses to reward. The task has three conditions: reward anticipation (anticipation of large win versus anticipation of no win), receipt of negative feedback (feedback of missed large win versus feedback of missed no win) and positive feedback (feedback of hit large win versus feedback of hit no win). A detailed description of the task is provided in Supplementary Appendix 1.

### Magnetic resonance imaging data acquisition

Structural and functional magnetic resonance imaging (MRI) data were acquired at eight Imagen assessment sites with 3-T MRI scanners of different manufacturers (Siemens, Munich, Germany; Philips, Best, The Netherlands; General Electric, Chalfont, St Giles, UK; Bruker, Ettlingen, Germany). The scanning variables were chosen to be compatible with all scanners. The same scanning protocol was used at all sites. In brief, high-resolution T1-weighted three-dimensional structural images were acquired for anatomical localization and co-registration with the functional time series. fMRI blood-oxygen-level dependent (BOLD) images were acquired with a gradient-echo, echo-planar imaging sequence. For the MID task, 300 volumes were acquired for each subject. Each volume consisted of 40 slices aligned to the anterior commissure−posterior commissure line (2.4 mm slice thickness, 1mm gap) acquired in a descending order. The echo time was optimized (echo time=30 m, repetition time=2200 ms) to provide reliable imaging of subcortical areas. See Supplementary Appendix 1 for detailed description of the fMRI data acquisition.

### Statistical analyses

#### Behavioral performance

We first tested whether participants were motivated by the potential of winning a reward. As a proxy of task engagement, we compared mean response accuracy in ‘no win' and ‘big win' trials using a paired-samples *t*-test (two-tailed), separately for participants with low and high ASD traits.

#### Imaging analyses

Imaging analyses were performed using the Statistical Parametric Mapping suite (SPM 8, Functional Imaging Laboratory, University College London, London, UK; www.fil.ion.ucl.ac.uk/spm). Analyses were performed at an *a priori* voxel threshold of *P<*0.01 and cluster threshold of *P<*0.05 with family-wise error (FWE) correction. Gender, handedness, site of scanning, WISC Verbal Comprehension and WISC Reasoning were included as covariates in all analyses, in line with previous studies.^35, 55, 56^ All findings are reported at whole-brain level. (NB: While SPM does not provide estimates of variance for between-groups brain activation data, we performed Levene's tests for equality of variances on extracted region-of-interest data (ROI; significant regions from whole-brain ANOVAs were extracted), which confirmed statistically equal variances between the groups.)

#### Effects of ASD traits and anxiety on reward processing

We ran a 2 × 2 ANOVA (ASD traits: low vs high; any anxiety: low (DAWBA bands 0/1) vs high (band 2 or above)) to test for main effects of ASD traits and anxiety, and an ASD-by-anxiety interaction, separately for reward anticipation, negative and positive feedback conditions. Where an ASD-by-anxiety interaction was found, we ran follow-up *t*-tests to assess between-groups differences in brain activations.

#### Covariates

We repeated the above ANOVAs twice, first adding ODD and then depression into the model as potential confounders.

#### Effects of emotional problems

To ensure that our main findings were not measure-specific, we ran confirmatory ANOVAs and *t*-tests using the SDQ emotional symptoms subscale (cut-off at 5) instead of DAWBA-defined ‘any anxiety'.

#### Longitudinal predictions

Our second aim was to assess whether the brain activations found in cross-sectional interaction analyses above are (a) a phenotypic manifestation of the co-occurrence between ASD traits and anxiety, or (b) whether they represent a biomarker that predicts not only cross-sectional, but also successive comorbidity between ASD traits and anxiety. To test this we conducted logistic regression analyses with anxiety (low vs high) at 2-year follow-up as the outcome, and brain activations found in our cross-sectional interaction effects as predictors of interest. Analyses were run in Stata 11,^57^ and the ROIs were extracted from the contrast maps for a given reward condition, using MarsBaR toolbox in SPM based on the WFUPickAtlas toolbox definitions.^58^ To ensure that the ROIs predicted new onset of anxiety, baseline anxiety status was added to the model as an additional predictor. We also added baseline ASD traits (continuous variable) to the model, to ensure that the predictions were not driven by the severity of ASD-specific impairments. To assess the specificity of brain predictions, regressions were run separately within low and high ASD trait groups. If an ROI predicted new-onset anxiety in one group but not the other, a regression model was run across the whole sample with an interaction term between ROI activations and ASD traits (low vs high) to identify the strength of a possible interaction. These analyses were not corrected for multiple comparisons since the ROIs were pre-defined based on our cross-sectional results.

## Results

### Participant characteristics

As expected, participants with high ASD traits scored significantly higher on all subscales of the DAWBA ASD section compared to those with low ASD traits (Table 1) and had a higher proportion of males. While the groups did not differ in age or performance IQ, youth with high ASD traits scored lower on verbal comprehension. Participants with high ASD traits scored higher on hyperactivity, emotional and conduct problems, and showed higher functional impairment. Those with high ASD traits were more likely to display symptoms of anxiety, depression and ODD. Sample characteristics were similar for the other two reward conditions (Supplementary Tables S1A-C).

### Behavioral performance

Paired-samples *t*-tests revealed that participants with high and low ASD traits both responded more accurately in ‘big win' compared to ‘no win' trials (high ASD traits: 65.1% vs 57.9%, *t*(69)=3.90, *P<*0.001; low ASD traits: 67.4% vs 53.8%, *t*(1401)=28.49, *P<*0.001), suggesting that both groups were motivated by the potential of winning a reward. The increase in response accuracy between ‘no win' and ‘big win' trials was higher for those with low vs high ASD traits, *t*(1470)=2.97, *P*=0.003, *d*=0.39.

### Reward anticipation

We first conducted an ANOVA to test the effects of ASD traits and anxiety on brain activations during reward anticipation.

#### Main effects

We found a main effect of ASD-trait severity and a main effect of anxiety (Table 2). Participants with high ASD traits (*n=*70) showed lower BOLD responses in the right superior frontal gyrus (SFG) extending to the anterior and midcingulate regions relative to youth with low ASD traits (*n=*1402). Participants with anxiety symptoms displayed increased activation in the right middle frontal and middle temporal gyri (MFG and MTG), irrespective of ASD-trait severity.

#### Interaction

**Figure 1:**
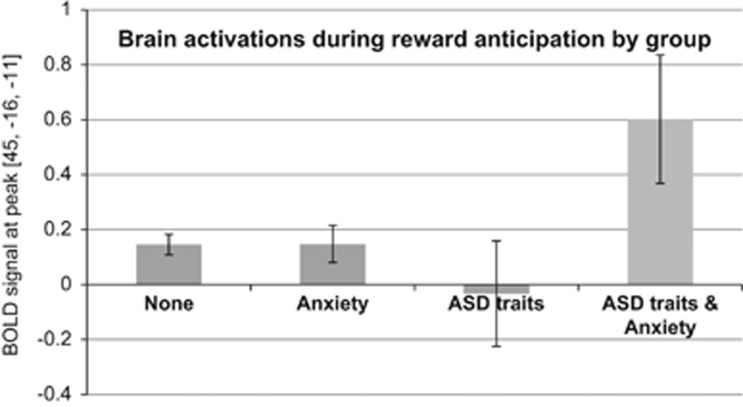
Interaction between autism spectrum disorder (ASD) traits and anxiety. Showing mean blood-oxygen-level dependent (BOLD) responses during reward anticipation and 95% confidence intervals at the cluster peak [45, -16, -11] located in the middle temporal gyrus. A similar pattern of results emerged for a peak in the right insula.

We also found an interaction between ASD traits and anxiety in a cluster encompassing right MTG, superior temporal gyrus and insula (Table 2). Figure 1 illustrates the interaction, showing significantly increased activation at the cluster's peak in ASD_ANX_ relative to all other groups. Follow-up *t*-tests revealed that the ASD_ANX_ (*n=*30) group showed significantly increased brain activation relative to ASD_ONLY_ (*n=*40) in the left insula and left inferior frontal gyrus (IFG), right MFG, as well as bilateral inferior parietal lobule and temporal areas. ASD_ANX_ also displayed increased activation in the left insula, left IFG and posterior brain regions relative to ANX_ONLY_ (*n=*326).

#### Covariates

All results remained significant after controlling for the effects of ODD, and we found two additional clusters in the ASD-by-anxiety interaction (ASD_ANX_>all other groups in the left IFG, insula and MFG). After controlling for the effects of depression, all results remained significant, except for one cluster in the ASD_ANX_>ASD_ONLY_ comparison (activations in the left insula and left IFG no longer significant).

#### Emotional problems

The results were broadly consistent with anxiety analyses and are described in detail in Supplementary Table S2A.

### Negative feedback

#### Main effects

We found a main effect of ASD-trait severity and a main effect of anxiety (Table 3). Participants with high ASD traits (*n=*78) showed lower activation in the right superior and medial frontal gyri relative to youth with low ASD traits (*n=*1523). Irrespective of ASD-trait severity, participants with anxiety symptoms (*n=*396) showed decreased activation in the following regions following negative feedback: bilateral caudate, bilateral IFG, right SFG, left MFG, left inferior parietal lobule and left MTG.

#### Interaction

**Figure 2:**
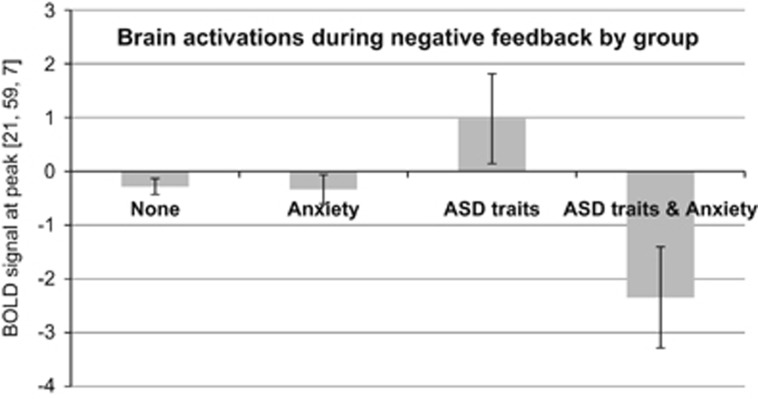
Interaction between autism spectrum disorder (ASD) traits and anxiety. Showing mean blood-oxygen-level dependent (BOLD) responses during negative reward feedback and 95% confidence intervals at cluster 1 peak [21, 59, 7] in the right superior frontal gyrus. The same pattern of results emerged for the two other clusters with peaks in the right caudate and left middle temporal gyrus, and middle frontal gyrus.

We also found an interaction between ASD traits and anxiety severity in the right caudate and putamen, prefrontal regions and left MTG. While anxiety did not affect brain activations in these regions in youth with low ASD traits, young people with ASD traits and anxiety showed markedly lower activations compared to ASD_ONLY_ (Figure 2). Follow-up *t*-tests revealed that ASD_ANX_ (*n=*35) had significantly decreased brain activation in bilateral MFG and IFG extending to the anterior cingulate, left precentral gyrus, and corpus callosum extending to the left caudate compared to ASD_ONLY_ (*n=*43). ASD_ANX_ also displayed decreased activation in bilateral caudate and putamen relative to ANX_ONLY_ (*n=*361).

#### Covariates

After controlling for the effects of ODD, the ASD-by-anxiety interaction remained significant, and we found an additional cluster encompassing the left caudate and left IFG. The main effect of anxiety remained significant in three out of six previously found clusters (the following clusters lost significance after controlling for ODD: right SFG *k*=120, *P*_FWE_=0.084; left MFG and IFG *k*=114, *P*_FWE_=0.104; left caudate and sgACC *k*=121, *P*_FWE_=0.081). The main effect of ASD was just below threshold for significance (frontal cluster *k*=131, *P*_FWE_=0.056). The ASD_ANX_< ANX_ONLY_ *t*-test remained significant, but 3/4 clusters in the ASD_ANX_<ASD_ONLY_ comparison lost significance (right MFG and IFG *k*=105, *P*_FWE_=0.084; left MFG and IFG *k*=109, *P*_FWE_=0.070; left IFG/rolandic operculum *k*=79, *P*_FWE_=0.260).

All results remained significant after controlling for the effects of depression, except for one cluster in the ASD_ANX_<ASD_ONLY_ comparison (activation in the left rolandic operculum no longer significant, *k*=107, *P*_FWE_=0.084). We also found two additional clusters encompassing the left IFG, left caudate and left putamen in the ASD-by-anxiety interaction.

#### Emotional problems

The results were broadly consistent with anxiety analyses and are described in detail in Supplementary Table S2B.

### Positive feedback

#### Main effects

Across the whole sample, we found a main effect of ASD-trait severity. Youth with high ASD traits (*n=*81) displayed increased activation in the bilateral thalamus and pallidum, as well as right putamen, compared to youth with low ASD traits (*n=*1645; Table 4). This finding remained significant after controlling for the effects of ODD, but lost significance after controlling for the effects of depression (right-sided cluster *P*_FWE_=0.061, *k*=148; left-sided cluster *P*_FWE_=0.671, *k*=65). No main effect of anxiety was found.

#### Interaction

We did not find a significant interaction.

#### Emotional problems

We did not find a main effect of ASD traits in our analyses with SDQ emotional subscale. Instead, we found a main effect of emotional problems in the left middle occipital gyrus (Supplementary Table S2C). No interaction was found.

### Longitudinal predictions

Finally, we investigated whether brain correlates of reward processing found in our interaction analyses, and relevant to the comorbid group, represent a mechanism underlying successive comorbidity between ASD traits and anxiety.

#### Reward anticipation

The following ROI predictors were tested (all right-sided): MTG, insula, Brodmann area (BA) 32 (dorsal cingulate), caudate, thalamus and medial frontal gyrus (medFG, defined using the ‘frontal_sup_medial' mask from the automatic anatomical labelling atlas in WFUPickAtlas).

High ASD traits: In participants with high ASD traits, increased activations in the right medFG and right BA 32 during reward anticipation were associated with a higher likelihood of anxiety at follow-up, with baseline anxiety and ASD traits included in the model (OR_medFGright_=62.33, 95% CI [1.46−2668.54], *P*=0.031; OR_BA32right_=33.22, 95% CI [1.47−750.36], *P*=0.028; Supplementary Table S3A).

Low ASD traits: None of the ROIs significantly predicted new-onset anxiety in participants with low ASD traits.

Interaction effects across the whole sample: The interaction term between ASD traits and right medFG was a significant predictor of new-onset anxiety at follow-up (OR=17.34, 95% CI (1.45−207.03), *P*=0.024). Likewise, the interaction between ASD traits and right BA 32 significantly predicted future anxiety (OR=15.75, 95% CI (1.32−187.48), *P*=0.029).

No significant results were found for the remaining ROIs.

#### Negative feedback

None of the ROIs we tested (right-sided: medFG, MFG, caudate, putamen, BA 32; left MTG) predicted dichotomous anxiety status at follow-up in either ASD traits group.

## Discussion

We found independent effects of ASD traits and anxiety on neural correlates of reward processing. We also found interaction effects whereby youth with combined ASD traits and anxiety showed distinctively high right MTG and insula activation when anticipating reward, and low prefrontal activation during negative feedback. Moreover, in participants with ASD traits, brain activation patterns during reward anticipation predicted new onset of anxiety 2 years later.

### ASD traits

During both reward anticipation and negative feedback, we observed attenuated BOLD activation in prefrontal regions in participants with high compared to low ASD traits. When anticipating reward, participants with high ASD traits showed reduced activation in dorsal ACC and right dorsal prefrontal cortex (PFC) (BA 9), regions involved in working memory, cognitive salience detection and monitoring of reward-based behavioral responses.^59, 60, 61^ Participants with ASD traits may attach less salience to rewards, consistent with previous studies showing reduced reward sensitivity in ASD youth.^17, 36^ During negative feedback, youth with ASD traits showed reduced activation in the right medial PFC compared to those with low ASD traits. Previous studies in healthy adults showed that while obtaining an expected reward is associated with an increase in medial PFC activation, reward omission leads to decreased activation in this region.^62, 63^ This functional modulation was proposed to reflect medial PFC's role in tracking rewarding outcomes. In this context, our results may indicate that participants with high ASD traits find the lack of expected reward relatively more punishing or aversive^64^ than those with low ASD traits. Combined with increased activation in reward-sensitive structures (putamen, thalamus, pallidum)^65^ in participants with high ASD traits following positive feedback, our results suggest that inadequate salience detection during reward anticipation may have led to exaggerated responses to both positive and negative reward outcomes.

### Anxiety

Participants with anxiety showed increased activation in the right MFG during reward anticipation, but decreased right MFG activation following negative feedback, compared to participants without anxiety. MFG is part of the lateral PFC,^66^ a region implicated in cognitive control via inhibition of prepotent behavioral responses.^67, 68, 69^ Increased activation in right MFG during anticipation suggests that participants with higher anxiety symptoms required more cognitive effort to maintain stimulus−reward representations active when faced with competing events.^68^ This is consistent with previous studies where anxious adolescents showed more emotional interference^70^ and heightened concern about making errors^23^ during reward processing compared to controls. However, we did not find the expected pattern of enhanced striatal activation during the anticipation of reward, which occurs specifically in social anxiety disorder.^71, 72^ This could relate to the low rate of social anxiety in our sample. Conversely to reward anticipation, following negative reward feedback, anxious participants displayed reduced activation in the lateral PFC (right MFG and SFG, bilateral IFG) and bilateral caudate.

### Interaction effects

The key aim of this study was to examine the interplay of ASD traits and anxiety symptoms during reward processing. We explored interaction effects to test whether the co-occurrence of ASD traits and anxiety was associated with a quantitative change in, or a qualitatively unique pattern of, reward-related brain activations. We found that youth with combined ASD traits and anxiety showed a unique pattern of high right insula activation during reward anticipation, as well as increased right MTG activation during anticipation and markedly low right-sided caudate, putamen, medial and lateral PFC activation during negative feedback. These effects remained significant after controlling for the effects of possible confounders (depression and ODD symptoms). Interestingly, insula hyperactivation was not observed in any of the main effects above, suggesting that youth with ASD traits and anxiety may display a qualitatively different pattern of neural activations during reward anticipation. The insula is implicated in ‘aversion-related' reward processing^59, 73^ particularly in anticipating and predicting salience of aversive events,^74, 75, 76, 77^ as well as in interoceptive processing.^78, 79, 80^ Interestingly, interoceptive prediction errors have been proposed to play a role in mood and anxiety disorders^78, 81^ and theory of mind.^82^ Future studies should test directly whether the distinct pattern of activations observed during reward anticipation in our ‘combined' group is related to interoceptive prediction errors, a possible etiological mechanism underlying the comorbidity between ASD and anxiety. Reduced right-sided caudate, putamen and medial PFC (BA 10) activation during negative feedback suggests that participants in the combined group may have found not receiving the reward more aversive than other participants, similarly to reward anticipation. We also found reduced lateral PFC activation in the combined group following negative feedback. Interestingly, some but not all activation patterns that characterize the interaction effect were also found in the main effect of ASD traits (BA 10) and anxiety (right lateral PFC and right caudate), suggesting shared and unique neural substrates of negative reward feedback in youth with combined ASD traits and anxiety.

### Longitudinal findings

In participants with high but not low ASD traits, increased right medFG and right dorsal cingulate activations during reward anticipation were associated with increased likelihood of anxiety symptoms 2 years later. Predictions were significant after controlling for baseline anxiety, showing that MRI can predict new onset of anxiety problems.

### Clinical implications

Our findings suggest that the presence of combined ASD traits and anxiety is associated with both a quantitatively potentiated neural response to negative reward feedback (interaction showing a further reduction in prefrontal activations found in main effects) as well as emergence of qualitatively different neural correlates during reward anticipation (activation in the right insula found exclusively in the interaction). This suggests that shared and distinct etiological mechanisms might be involved in the comorbidity between ASD and anxiety, and, if replicated, carries important clinical implications. If the co-occurrence of anxiety in ASD is underpinned by a distinct pathophysiological mechanism, the comorbid group may need to be recognized as a distinct nosological category and be researched in its own right. Moreover, it is possible that medication response in this group is also different. We know already from ADHD literature that the effectiveness of methylphenidate is reduced in some youth with ASD^83^ and in those with comorbid ADHD and anxiety.^84, 85, 86^ In addition, a specific biomarker of anxiety in ASD could aid differential diagnosis in cases where comorbid anxiety may be phenomenologically indistinguishable from ASD.^87^

Second, although MRI findings predicted only a small portion of the variance in new onset of anxiety at follow-up, brain activations were a significant predictor that could be used in establishing useful biomarkers of anxiety risk in youth with ASD traits. Our design strengthens the implication that the pattern of right-sided medFG and dorsal cingulate activations during reward processing is not merely a marker of anxiety, but may reflect an underlying mechanism by which young people with ASD traits become anxious. Ultimately, finding an MRI biomarker of anxiety in ASD has a potential of guiding treatment interventions and measuring treatment response, which is especially useful in cases where the value of clinical interview is limited due to social communication difficulties.^5^

Third, recent evidence suggests that disrupted processing of reward may lead to decision-making problems.^88^ Future studies should investigate whether reward-processing deficits can explain the presence of executive function deficits in ASD,^89^ and explore the role of comorbid anxiety in the process.

### Limitations

Although we investigated both anticipation and feedback phases of reward processing, task learning was performed outside of the scanner; therefore it was not possible to study the neural correlates of stimulus−reward learning. Second, due to sample size limitations we did not distinguish between different types of anxiety disorders. Future studies should test whether the differential impact of specific types of anxiety on reward processing, seen in typically developing youth,^90^ holds in youth with ASD traits. Third, while our *a priori* choice of a cluster-forming threshold of *P<*0.01 at the voxel level is in line with previous reward-processing fMRI studies in adolescents that used the same^91^ or more lenient^92^ voxel-wise thresholds, and a large sample size is likely to limit the rate of false positives,^41^ it is still important that our results are replicated in an independent sample. Finally, the relative contribution of anxiety and ASD-specific difficulties to reward processing in youth with a clinical diagnosis of ASD remains to be studied.

In conclusion, over and above the independent effects of ASD traits and anxiety, we found qualitatively distinct and quantitatively potentiated neural correlates of reward processing in youth with combined anxiety and ASD traits. Future studies should assess whether the apparent co-occurrence of ASD and anxiety is associated with distinct etiological mechanisms.

## Figures and Tables

**Table 1:** Demographic characteristics of the sample (mean±s.d. or frequency data, showing results for participants with scans available for the reward anticipation condition)

	*Low ASD traits*	*High ASD traits*
*Baseline*		
* n*	1402	70
Male gender	657 (46.9%)	47 (67.1%)**
Age in years	14.4±0.4	14.4±0.4
** ** WISC Verbal	112.0±14.7	108.1±15.1*
** ** WISC Reasoning	108.1±13.9	108.8±14.4
		
ASD symptoms (DAWBA)		
** ** Total	0.3±1.6	17.4±5.5***
** ** Social difficulties	0.2±1.2	9.9±4.1***
** ** Repetitive behaviors	0.1±0.5	6.8±3.7***
** ** Language development	0.1±0.3	0.6±0.9***
		
Continuous psychopathology (SDQ)		
** ** Emotional symptoms	1.8±1.9	3.5±2.9***
** ** Conduct problems	1.6±1.5	2.9±2.2***
** ** Hyperactivity	2.8±2.2	4.5±2.9***
** ** Impact	0.6±1.3	2.2±2.4***
		
Diagnostic categories		
** ** Any anxiety	326 (23.3%)	30 (42.9%)***
** ** Depression	44 (3.1%)	10 (14.3%)***
** ** ODD	483 (34.5%)	43 (61.4%)***
		
*2-year follow-up*		
* n*	1019	50
Male gender	465 (45.7%)	35 (70.0%)**
		
SDQ		
** ** Emotional symptoms	1.6±1.9	2.9±2.9**
** ** Conduct problems	1.4±1.4	2.0±1.9*
** **Hyperactivity	2.2±2.0	3.6±2.5***
** **Impact	0.5±1.4	1.3±2.1*
		
Diagnostic categories		
** ** Any anxiety	196 (19.3%)	17 (34.0%)*
** ** Depression	36 (3.5%)	6 (12.0%)*
** ** ODD	295 (29.0%)	23 (46.0%)*

Abbreviations: ASD, autism spectrum disorder; DAWBA, Development and Well-Being Assessment; ODD, oppositional defiant disorder; SDQ, Strengths and Difficulties Questionnaire.

*P<0.05; **P<0.01; ***P<0.001 (*t*-test or chi-square).

**Table 2:** The effects of ASD traits and anxiety on BOLD responses during reward anticipation

			*Peak MNI coordinates*				
*Region*	*Brodmann area*	*Cluster size (voxels)*	x	y	z	Z	P *(FWE)*
							
*Interaction: ASD traits x anxiety*							
R middle and superior temporal gyri, R insula	21/22/13	283	45	−16	−11	3.97	0.001
			57	−37	−8	3.80	
			63	−16	−11	3.71	
							
*High<low ASD traits*							
R superior and medial frontal gyri extending bilaterally to the dACC and MCC	9/24/32	936	−18	17	37	4.88	<0.001
			18	17	49	4.72	
			9	50	37	4.58	
							
*Any anxiety>no anxiety*							
R middle frontal gyrus	8	254	39	23	43	4.41	0.002
			21	59	37	3.89	
			42	8	49	3.66	
R middle temporal gyrus	21	209	45	−16	−11	3.98	0.008
			60	−16	−5	3.67	
			57	−37	−11	3.47	
							
*ASD*_*ANX*_*>ASD*_*ONLY*_							
R middle temporal gyrus	21	257	57	−37	−8	4.56	<0.001
			63	−13	−2	4.31	
			69	−37	−2	3.90	
R middle frontal gyrus	8	251	42	23	43	4.27	<0.001
			42	11	52	3.71	
			42	32	43	3.45	
L insula, L inferior frontal gyrus	13/45/47	162	−42	17	−11	4.27	0.011
			−36	17	1	3.43	
			−45	17	7	3.36	
L inferior parietal lobule	40	124	−57	−43	55	3.86	0.047
			−48	−61	52	3.33	
			−66	−37	37	3.11	
L superior and middle temporal gyri	39/22/40	246	−60	−67	7	3.71	0.001
			−51	−82	7	3.70	
			−51	−79	22	3.68	
R inferior parietal lobule	40	256	51	−52	55	3.60	<0.001
			60	−34	55	3.49	
			48	−34	64	3.41	
							
*ASD*_*ANX*_*>ANX*_*ONLY*_							
L insula, L inferior frontal gyrus	47	186	−42	17	−8	4.68	0.017
			−36	14	4	3.65	
			−54	20	1	3.57	
L and R cuneus and calcarine	18/17	222	−12	−82	10	4.30	0.006
			12	−85	10	3.77	
			−6	−76	22	3.38	

Abbreviations: ANX_ONLY_, high anxiety and low ASD traits; ASD, autism spectrum disorder; ASD_ANX_, high ASD traits and anxiety; ASD_ONLY_, high ASD traits, low anxiety; BOLD, blood-oxygen-level dependent; dACC, dorsal anterior cingulate cortex; FWE, family-wise error correction; L, left hemisphere; MCC, middle cingulate cortex; MNI, Montreal Neurological Institute; R, right hemisphere.

**Table 3:** The effects of ASD traits and anxiety on BOLD responses during negative reward feedback

			*Peak MNI coordinates*				
*Region*	*Brodmann area*	*Cluster size (voxels)*	x	y	z	Z	P *(FWE)*
*Interaction: ASD traits x anxiety*							
R superior and medial frontal gyri	10	223	21	59	7	4.95	0.003
			27	59	1	4.84	
			18	47	22	4.34	
R caudate, R putamen, R middle and inferior frontal gyri	10	301	21	23	1	4.73	<0.001
			27	38	−5	4.46	
			21	23	13	3.76	
L middle temporal gyrus		183	−51	−76	13	4.05	0.010
			−57	−70	10	3.87	
			−24	−85	16	3.36	
							
*High<Low ASD traits*							
R superior and medial frontal gyri	10	137	21	44	1	4.33	0.046
			18	53	13	4.06	
			30	56	−2	3.71	
							
*Any anxiety<no anxiety*							
R superior frontal gyrus extending to R medial frontal gyrus	10	145	21	59	7	5.08	0.034
			21	62	19	3.62	
			6	65	28	2.75	
R caudate, R inferior frontal gyrus		449	18	47	22	4.79	<0.001
			21	23	1	4.67	
			27	38	−5	4.29	
L inferior parietal lobule		184	−36	−46	28	4.62	0.009
			−24	−58	25	4.53	
			−24	−52	34	3.87	
L middle temporal gyrus		182	−60	−70	7	4.12	0.010
			−51	−76	13	3.85	
			−51	−67	13	3.33	
L middle and inferior frontal gyrus		193	−48	29	−5	4.04	0.007
			−30	44	22	3.97	
			−30	41	14	3.72	
L caudate extending to the subgenual ACC	24/25	144	−9	23	−5	3.71	0.036
			−18	23	4	3.67	
			−12	14	4	3.41	
							
*ASD*_*ANX*_*<ASD*_*ONLY*_							
R middle and inferior frontal gyri (extending to the midcingulate/ACC)	46/32	143	27	32	31	4.30	0.015
			33	38	16	4.12	
			42	35	13	3.66	
L inferior and middle frontal gyri	46	136	−48	29	−2	4.20	0.020
			−36	26	13	3.39	
			−45	32	19	3.38	
L rolandic operculum / precentral gyrus, L inferior frontal gyrus	22	120	−63	5	4	4.00	0.040
			−54	2	7	3.65	
			−57	14	7	3.55	
L and R lateral ventricles, corpus callosum (extending to L caudate)		179	9	−10	22	3.60	0.004
			−6	−19	25	3.35	
			−1	25	3.35		
							
*ASD*_*ANX*_*<ANX*_*ONLY*_							
R putamen and R caudate extending to subcallosal gyrus / gyrus rectus, R superior and medial frontal gyri	34/13/10	472	27	59	1	5.33	<0.001
			15	56	7	4.70	
			18	20	1	3.89	
L putamen and L caudate, L middle frontal gyrus	47	227	−30	44	−8	4.17	0.003
			−12	11	1	4.00	
			−21	23	1	3.88	

Abbreviations: ACC, anterior cingulate cortex; ASD, autism spectrum disorder; ANX_ONLY_, high anxiety and low ASD traits; ASD_ANX_, high ASD traits and anxiety; ASD_ONLY_, high ASD traits and low anxiety; BOLD, blood-oxygen-level dependent; FWE, family-wise error correction; L, left hemisphere; MNI, Montreal Neurological Institute; R, right hemisphere.

**Table 4:** The effects of ASD traits and anxiety on brain activation patterns following positive feedback

			*Peak MNI coordinates*				
*Region*	*Brodmann area*	*Cluster size (voxels)*	x	y	z	Z	P *(FWE)*
*High>low ASD traits*							
R thalamus, R putamen, R globus pallidus		163	30	−4	4	5.03	0.039
			24	−10	4	4.78	
			21	−22	7	3.74	
L thalamus, L globus pallidus, midbrain, extending to L hippocampus		185	15	−16	−5	4.23	0.021
			−15	−7	1	4.21	
			−9	22	1	3.66	

Abbreviations: ASD, autism spectrum disorder; FWE, family-wise error correction; L, left hemisphere; MNI, Montreal Neurological Institute; R, right hemisphere.
