# Supplementary material for: Disentangling the autism−anxiety overlap: fMRI of reward processing in a community-based longitudinal study
Source: Transl Psychiatry. 2016 Jun 28;6(6):e845–. doi: 10.1038/tp.2016.107 (PMC4931605; doi:10.1038/tp.2016.107)
Supplement: Supplementary Information [file tp2016107x1.docx]

**SUPPLEMENTARY INFORMATION**

**Appendix 1. Methods and sample characteristics.**

***Modified Monetary Incentive Delay Task***

The modified Monetary Incentive Delay task used in the present study was an adaptation of the task from Knutson et al (1). This event-related task consisted of 66 ten-second trials. In each particular trial, participants were presented with one of three cue shapes (cue, 250 ms) denoting whether a target (a white square) would subsequently appear on the left or right side of the screen and whether 0, 2 or 10 points could be won in that particular trial (Figure S1). After a variable delay (4000-4500 ms), participants were instructed to respond by pressing a button with their left or right index finger as soon as the target appeared. Feedback on whether and how many points were won during the trial was presented for 1450 ms after the response. Using a tracking algorithm, task difficulty (i.e., target duration varied between 250 and 400 ms) was individually adjusted such that each participant responded successfully on ~66% of trials. Participants had first completed a practice session outside the scanner (for ~5 minutes), during which they were instructed that after the scanning session concluded, the points won on the task would be converted into chocolate candies (one small candy for every 5 points). Functional magnetic resonance imaging (MRI), blood oxygen-level dependent (BOLD)-responses were measured during reward anticipation and reward feedback. Task presentation and recording of the behavioral responses were performed using Visual Basic 2005 with .NET Framework Version 2.0, and the visual and response grip system from Nordic Neuro Lab (NordicNeuroLab AS, Bergen, Norway).


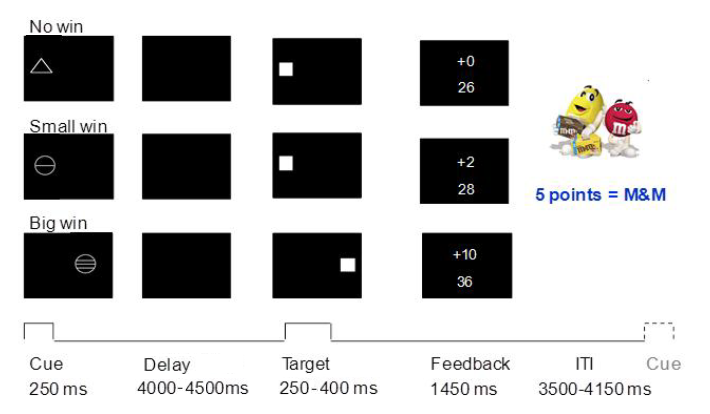


**Figure S1.** Outline of the stages of the modified Monetary Incentive Delay task.

***Magnetic Resonance Imaging Data Acquisition***

Structural and functional MRI data were acquired at eight Imagen assessment sites with 3T MRI scanners of different manufacturers (Siemens, Munich, Germany; Philips, Best, The Netherlands; General Electric, Chalfont St Giles, UK; Bruker, Ettlingen, Germany). A key challenge for the ability to pool data acquired on MR scanners of different manufacturers relates to their variation in availability and implementation of particular image-acquisition techniques. To address this problem, for each technique, a set of parameters compatible with all scanners, particularly those directly affecting image contrast or signal-to-noise, was devised and held constant across sites. Where manufacturer-specific choices had to be made (for example the design of head coil), the best manufacturer-specific option was used at all sites with the same scanner type. Two quality control procedures are regularly implemented at each site: (1) The American College of Radiology phantom is scanned to provide information about geometric distortions and signal uniformity related to hardware differences in radiofrequency coils and gradient systems, image contrast and temporal stability, and a custom phantom58 is scanned for diffusion-related parameters. (2) Several healthy volunteers are regularly scanned at each site to assess factors that cannot be measured using phantoms alone and at multiple sites to determine inter-site variability in structural and functional measures (for example, tissue contrast in raw MRI signal, tissue relaxation properties). More details about quality information can be found in Schumann et al. (2).

The scanning variables were specifically chosen to be compatible with all scanners. The same scanning protocol was used at all sites. High-resolution T1-weighted three-dimensional structural images were acquired for anatomical localization and co-registration with the functional time series. The T1-weighted magnetization prepared gradient echo sequence (MPRAGE) images were obtained using a modified protocol based on the ADNI project (<http://adni.loni.ucla.edu/methods/documents/mri-protocols/>). The images comprised 160 slices with 1.1 × 1.1 × 1.1 mm^3^ voxel size, field of view frequency 28 cm. Functional MRI BOLD images were acquired with a gradient-echo, echo-planar imaging sequence. For the modified Monetary Incentive Delay task, 300 volumes were acquired for each subject. Each volume consisted of 40 slices aligned to the anterior commissure- posterior commissure line (2.4mm slice thickness, 1mm gap) acquired in a descending order. The echo time was optimized (echo time = 30 msec, repetition time = 2200 msec) to provide reliable imaging of subcortical areas.

Functional MRI data were pre-processed and analyzed with SPM8 (Statistical Parametric Mapping; <http://www.fil.ion.ucl.ac.uk/spm>). Time series data were slice-time corrected using the first slice as the reference for interpolation, and then corrected for movement (spatial realignment) to the first volume. Time series data were then non-linearly warped on the MNI space, using a custom EPI template and smoothed with a Gaussian kernel of 5mm Full-Width Half Maximum (FWHM).

At the first level of analysis, the model contained the onset of each cue and each feedback presentation. This enables separate analyses of reward anticipation and reward feedback conditions. Each trial (e.g., reward feedback large win) was convolved using the SPM default Hemodynamic Response Function (HRF). Estimated movement parameters were added to the design matrix in the form of 18 additional columns (3 translations, 3 rotations, 3 quadratic and 3 cubic translations, and each 3 translations with a shift of ±1 TR). For the current analyses, we were interested in the following contrasts: “anticipation of large win versus anticipation of no win”, “feedback of missed large win versus feedback of missed no win”, and “feedback of hit large win versus feedback of hit no win”.

**Table S1A.** Demographic characteristics of the sample for participants with scans available for the reward anticipation condition.

|  | **None** | **Anxiety only** | **ASD traits only** | **ASD traits & anxiety** |
| --- | --- | --- | --- | --- |
|  |  |  |  |  |
| **Baseline** |  |  |  | a, b, c, d = different letters indicate a significant group difference from each other at *p*<.05 (Bonferroni corrected).  *DAWBA*, Development and Well-Being Assessment.  *SDQ*, Strengths and Difficulties Questionnaire. |
| **n** | 1076 | 326 | 40 | 30 |
| **Male gender** | 527 (49.0%)^a^ | 130 (39.9%)^b^ | 28 (70.0%)^c^ | 19 (63.3%)^a^ |
| **Age in years** | 14.4 ± 0.4 | 14.4 ± 0.4 | 14.4 ± 0.4 | 14.5 ± 0.4 |
| WISC Verbal | 112.8 ± 14.6^a^ | 109.2 ± 14.7^b^ | 110.2 ± 16.2^a,b^ | 105.3 ± 13.4^b^ |
| WISC Reasoning | 108.6 ± 13.9^a^ | 106.5 ± 13.8^b^ | 113.3 ± 13.4^a^ | 102.9 ± 13.7^b^ |
| **ASD symptoms (DAWBA)** |  |  |  |  |
| total | 0.3 ± 1.2^a^ | 0.6 ± 2.4^b^ | 16.6 ± 5.3^c^ | 18.4 ± 5.6^d^ |
| social difficulties | 0.1 ± 0.8^a^ | 0.4 ± 1.9^b^ | 9.4 ± 3.9^c^ | 10.7 ± 4.4^d^ |
| repetitive behaviors | 0.1 ± 0.5^a^ | 0.1 ± 0.6^a^ | 6.5 ± 3.4^b^ | 7.3 ± 4.0^c^ |
| language development | 0.1 ± 0.3^a^ | 0.1 ± 0.3^a^ | 0.7 ± 1.0^b^ | 0.4 ± 0.8^c^ |
| **Continuous psychopathology (SDQ)** |  |  |  |  |
| emotional symptoms | 1.2 ± 1.3^a^ | 3.9 ± 2.0^b^ | 2.0 ± 1.9^c^ | 5.5 ± 2.8^d^ |
| conduct problems | 1.4 ± 1.4^a^ | 2.0 ± 1.8^b^ | 2.3 ± 1.9^b^ | 3.8 ± 2.3^c^ |
| hyperactivity | 2.6 ± 2.1^a^ | 3.5 ± 2.4^b^ | 3.5 ± 2.4^b^ | 5.9 ± 3.1^c^ |
| impact | 0.4 ± 0.9^a^ | 1.2 ± 1.8^b^ | 1.0 ± 1.6^b^ | 3.8 ± 2.6^c^ |
| **Diagnostic categories** |  |  |  |  |
| any anxiety | 0^a^ | 326 (100%)^b^ | 0^a^ | 30 (100%)^b^ |
| depression | 16 (1.5%)^a^ | 28 (8.6%)^b^ | 2 (5.0%)^a,b^ | 8 (26.7%)^c^ |
| ODD | 301 (28.0%)^a^ | 182 (55.8%)^b^ | 23 (57.5%)^b^ | 20 (66.7%)^b^ |
| **2-year follow up** |  |  |  |  |
| **n** | 793 | 226 | 28 | 22 |
| **Male gender** | 380 (48.0%)^a^ | 85 (37.6%)^b^ | 21 (75.0%)^c^ | 14 (63.6%)^a,b,c^ |
| **Continuous psychopathology (SDQ)** |  |  |  |  |
| emotional symptoms | 1.2 ± 1.5^a^ | 3.1 ± 2.3^b^ | 1.6 ± 2.2^a^ | 4.5 ± 3.0^c^ |
| conduct problems | 1.3 ± 1.4^a^ | 1.7 ± 1.6^b^ | 1.6 ± 1.9^a,b^ | 2.5 ± 1.9^b^ |
| hyperactivity | 2.1 ± 1.9^a^ | 2.6 ± 2.1^b^ | 2.8 ± 2.2^a,b^ | 4.7 ± 2.5^c^ |
| impact | 0.4 ± 1.2^a^ | 1.1 ± 1.9^b^ | 1.0 ± 2.0^a,b^ | 1.6 ± 2.3^b,c^ |
| **Diagnostic categories** |  |  |  |  |
| any anxiety | 96 (12.1%)^a^ | 100 (44.2%)^b^ | 5 (17.9%)^c^ | 12 (54.5%)^b^ |
| depression | 18 (2.3%)^a^ | 18 (8.0%)^b^ | 2 (7.1%)^b^ | 4 (18.2%)^b^ |
| ODD | 201 (25.4%)^a^ | 94 (41.8%)^b^ | 10 (35.7%)^b^ | 13 (59.1%)^b^ |

**Table S1B.** Demographic characteristics of the sample for participants with scans available for the negative reward feedback condition.

**p* < .05;

***p* < .01;

****p* < .001.

a, b, c, d = different letters indicate a significant group difference from each other at *p*<.05 (Bonferroni corrected).

|  | **Total: Low ASD traits** | **Total: High ASD traits** | **None** | **Anxiety only** | **ASD traits only** | **ASD traits & anxiety** |
| --- | --- | --- | --- | --- | --- | --- |
|  |  |  |  |  |  |  |
| **Baseline** |  |  |  |  |  |  |
| **n** | 1523 | 78 | 1162 | 361 | 43 | 35 |
| **Male gender** | 738 (48.5%) | 54 (69.2%)*** | 595 (51.2%)^a^ | 143 (39.6%)^b^ | 30 (69.8%)^a^ | 24 (68.6%)^a^ |
| **Age in years** | 14.4 ± 0.4 | 14.4 ± 0.4 | 14.4 ± 0.4 | 14.4 ± 0.4 | 14.4 ± 0.4 | 14.5 ± 0.4 |
| WISC Verbal | 111.2 ± 14.7 | 107.0 ± 15.9* | 112.0 ± 14.6^a^ | 108.8 ± 15.0^b^ | 109.5 ± 16.0^a,b^ | 104.0 ± 15.5^b^ |
| WISC Reasoning | 107.7 ± 14.2 | 107.2 ± 15.6 | 108.3 ± 14.0^a^ | 105.9 ± 14.6^b^ | 112.6 ± 14.4^a^ | 100.6 ± 14.2^b^ |
| **ASD symptoms (DAWBA)** |  |  |  |  |  |  |
| total | 0.3 ± 1.6 | 17.1 ± 5.5*** | 0.3 ± 1.3^a^ | 0.6 ± 2.4^b^ | 16.3 ± 5.5^c^ | 18.2 ± 5.3^d^ |
| social difficulties | 0.2 ± 1.2 | 9.1 ± 4.2*** | 0.1 ± 0.8^a^ | 0.4 ± 1.9^b^ | 9.4 ± 4.2^c^ | 10.4 ± 4.2^d^ |
| repetitive behaviors | 0.1 ± 0.6 | 6.6 ± 3.6*** | 0.1 ± 0.5^a^ | 0.1 ± 0.7^a^ | 6.1 ± 3.3^b^ | 7.2 ± 3.9^c^ |
| language development | 0.1 ± 0.3 | 0.6 ± 0.9*** | 0.1 ± 0.3^a^ | 0.1 ± 0.3^a^ | 0.7 ± 1.0^b^ | 0.5 ± 0.9^c^ |
| **Continuous psychopathology (SDQ)** |  |  |  |  |  |  |
| emotional symptoms | 1.9 ± 1.9 | 3.5 ± 2.9*** | 1.2 ± 1.3^a^ | 4.0 ± 2.1^b^ | 1.8 ± 1.9^a^ | 5.6 ± 2.6^c^ |
| conduct problems | 1.6 ± 1.6 | 2.9 ± 2.1*** | 1.4 ± 1.4^a^ | 2.1 ± 1.8^b^ | 2.3 ± 1.8^b^ | 3.7 ± 2.3^c^ |
| hyperactivity | 2.8 ± 2.2 | 4.6 ± 2.9*** | 2.6 ± 2.1^a^ | 3.6 ± 2.4^b^ | 3.5 ± 2.3^b^ | 6.1 ± 2.8^c^ |
| impact | 0.6 ± 1.3 | 2.1 ± 2.4*** | 0.4 ± 1.0^a^ | 1.3 ± 1.9^b^ | 0.9 ± 1.5^b^ | 3.5 ± 2.5^c^ |
| **Diagnostic categories** |  |  |  |  |  |  |
| any anxiety | 361 (23.7%) | 35 (44.9%)*** | 0^a^ | 361 (100%)^b^ | 0^a^ | 35 (100%)^b^ |
| depression | 48 (3.2%) | 13 (16.7%)*** | 20 (1.7%)^a^ | 28 (7.8%)^b^ | 3 (7.0%)^a,b^ | 10 (28.6%)^c^ |
| ODD | 534 (35.1%) | 49 (62.8%)*** | 326 (28.1%)^a^ | 208 (57.6%)^b^ | 25 (58.1%)^b^ | 24 (68.6%)^b^ |
| **2-year follow up** |  |  |  |  |  |  |
| **n** | 1124 | 55 | 872 | 252 | 30 | 25 |
| **Male gender** | 533 (47.4%) | 37 (67.3%)** | 437 (50.1%)^a^ | 96 (38.1%)^b^ | 21 (70.0%)^a^ | 16 (64.0%)^a^ |
| **Continuous psychopathology (SDQ)** |  |  |  |  |  |  |
| emotional symptoms | 1.7 ± 1.9 | 3.0 ± 3.0** | 1.3 ± 1.6^a^ | 2.9 ± 2.3^b^ | 1.8 ± 2.4^a^ | 4.4 ± 3.0^c^ |
| conduct problems | 1.4 ± 1.4 | 2.2 ± 2.0** | 1.3 ± 1.4^a^ | 1.6 ± 1.6^b^ | 1.9 ± 2.1^a,c^ | 2.5 ± 1.9^c^ |
| hyperactivity | 2.3 ± 2.0 | 3.8 ± 2.5*** | 2.2 ± 1.9^a^ | 2.7 ± 2.1^b^ | 3.0 ± 2.2^a,b^ | 4.7 ± 2.4^c^ |
| impact | 0.5 ± 1.3 | 1.4 ± 2.2** | 0.4 ± 1.1^a^ | 1.0 ± 1.8^b^ | 1.2 ± 2.2^b^ | 1.6 ± 2.3^b^ |
| **Diagnostic categories** |  |  |  |  |  |  |
| any anxiety | 228 (20.3%) | 21 (38.2%)** | 116 (13.3%)^a^ | 112 (44.4%)^b^ | 7 (23.3%)^a,b^ | 14 (56.0%)^b^ |
| depression | 38 (3.4%) | 7 (12.7%)** | 20 (2.3%)^a^ | 18 (7.1%)^b^ | 3 (10.0%)^b^ | 4 (16.0%)^b^ |
| ODD | 322 (28.7%) | 26 (47.3%)** | 219 (25.1%)^a^ | 103 (40.9%)^b^ | 12 (40.0%)^a,b^ | 14 (56.0%)^b^ |

**Table S1C.** Demographic characteristics of the sample for participants with scans available for the positive reward feedback condition.

**p* < .05;

***p* < .01;

****p* < .001.

a, b, c, d = different letters indicate a significant group difference from each other at *p*<.05 (Bonferroni corrected).

|  | **Total: Low ASD traits** | **Total: High ASD traits** | **None** | **Anxiety only** | **ASD traits only** | **ASD traits & anxiety** |
| --- | --- | --- | --- | --- | --- | --- |
|  |  |  |  |  |  |  |
| **Baseline** |  |  |  |  |  |  |
| **n** | 1645 | 81 | 1256 | 389 | 44 | 37 |
| **Male gender** | 795 (48.3%) | 57 (70.4%)*** | 639 (50.9%)^a^ | 156 (40.1%)^b^ | 32 (72.7%)^c^ | 25 (67.6%)^a,c^ |
| **Age in years** | 14.4 ± 0.4 | 14.4 ± 0.4 | 14.4 ± 0.4 | 14.4 ± 0.4 | 14.4 ± 0.4 | 14.5 ± 0.4 |
| WISC Verbal | 111.3 ± 14.8 | 107.2 ± 15.5* | 112.2 ± 14.6^a^ | 108.5 ± 15.0^b^ | 110.1 ± 15.4^a,b^ | 103.8 ± 15.1^b^ |
| WISC Reasoning | 107.5 ± 14.1 | 107.5 ± 14.9 | 108.0 ± 14.0^a^ | 105.8 ± 14.4^b^ | 113.0 ± 13.2^a^ | 101.1 ± 14.5^b^ |
| **ASD symptoms (DAWBA)** |  |  |  |  |  |  |
| total | 0.3 ± 1.5 | 17.4 ± 5.3*** | 0.3 ± 1.2^a^ | 0.6 ± 2.2^b^ | 16.8 ± 5.4^c^ | 18.0 ± 5.2^d^ |
| social difficulties | 0.2 ± 1.1 | 10.0 ± 4.1*** | 0.1 ± 0.8^a^ | 0.4 ± 1.8^b^ | 9.7 ± 4.0^c^ | 10.3 ± 4.2^c^ |
| repetitive behaviors | 0.1 ± 0.5 | 6.8 ± 3.6*** | 0.1 ± 0.5^a^ | 0.1 ± 0.6^a^ | 6.4 ± 3.4^b^ | 7.2 ± 3.8^c^ |
| language development | 0.1 ± 0.3 | 0.6 ± 0.9*** | 0.1 ± 0.3^a^ | 0.1 ± 0.3^a^ | 0.7 ± 1.0^b^ | 0.5 ± 0.9^b^ |
| **Continuous psychopathology (SDQ)** |  |  |  |  |  |  |
| emotional symptoms | 1.9 ± 1.9 | 3.5 ± 2.9*** | 1.2 ± 1.3^a^ | 3.9 ± 2.1^b^ | 1.9 ± 1.9^c^ | 5.5 ± 2.6^d^ |
| conduct problems | 1.6 ± 1.6 | 3.0 ± 2.2*** | 1.5 ± 1.4^a^ | 2.1 ± 1.8^b^ | 2.3 ± 1.8^b^ | 3.8 ± 2.3^c^ |
| hyperactivity | 2.9 ± 2.2 | 4.7 ± 2.9*** | 2.6 ± 2.1^a^ | 3.6 ± 2.4^b^ | 3.6 ± 2.4^b^ | 6.1 ± 2.9^c^ |
| impact | 0.6 ± 1.3 | 2.2 ± 2.5*** | 0.4 ± 1.0^a^ | 1.2 ± 1.9^b^ | 0.9 ± 1.5^a,b^ | 3.7 ± 2.7^c^ |
| **Diagnostic categories** |  |  |  |  |  |  |
| any anxiety | 389 (23.6%) | 37 (45.7%)*** | 0 | 389 (100%) | 0 | 37 (100%) |
| depression | 51 (3.1%) | 12 (14.8%)*** | 21 (1.7%)^a^ | 30 (7.7%)^b^ | 2 (4.5%)^a,b^ | 10 (27.0%)^c^ |
| ODD | 577 (35.1%) | 51 (63.0%)*** | 357 (28.4%)^a^ | 220 (56.6%)^b^ | 25 (56.8%)^b^ | 26 (70.3%)^b^ |
| **2-year follow up** |  |  |  |  |  |  |
| **n** | 1191 | 55 | 925 | 266 | 29 | 26 |
| **Male gender** | 561 (47.1%) | 39 (70.9%)** | 460 (49.7%)^a^ | 101 (38.0%)^b^ | 22 (75.9%)^c^ | 17 (65.4%)^a,c^ |
| **Continuous psychopathology (SDQ)** |  |  |  |  |  |  |
| emotional symptoms | 1.7 ± 1.9 | 3.0 ± 3.0** | 1.3 ± 1.6^a^ | 2.9 ± 2.3^b^ | 1.5 ± 2.2^a^ | 4.5 ± 3.0^c^ |
| conduct problems | 1.4 ± 1.4 | 2.1 ± 2.0* | 1.3 ± 1.4^a^ | 1.7 ± 1.6^b^ | 1.7 ± 1.9^a,b,c^ | 2.6 ± 2.0^c^ |
| hyperactivity | 2.3 ± 2.0 | 3.8 ± 2.5*** | 2.2 ± 1.9^a^ | 2.7 ± 2.1^b^ | 2.9 ± 2.1^a,b^ | 4.9 ± 2.5^c^ |
| impact | 0.5 ± 1.4 | 1.3 ± 2.1** | 0.4 ± 1.1^a^ | 1.0 ± 1.9^b^ | 1.0 ± 2.0^a,b^ | 1.7 ± 2.3^b^ |
| **Diagnostic categories** |  |  |  |  |  |  |
| any anxiety | 235 (19.7%) | 19 (34.5%)* | 120 (13.0%)^a^ | 115 (43.2%)^b^ | 5 (17.2%)^a^ | 14 (53.8%)^b^ |
| depression | 43 (3.6%) | 6 (10.9%)* | 24 (2.6%)^a^ | 19 (7.1%)^b^ | 2 (6.9%)^a,b^ | 4 (15.4%)^b^ |
| ODD | 338 (28.4%) | 25 (45.5%)** | 230 (24.9%)^a^ | 108 (40.8%)^b^ | 10 (34.5%)^a,b^ | 15 (57.7%)^b^ |

**Table S1D.** Number of participants with ASD traits who met criteria for DSM-IV defined diagnoses based on DAWBA computer predictions at probability band 2 or higher.

| **Diagnostic category** | **Anticipation (n=70)** | **Negative feedback (n=78)** | **Positive feedback (n=81)** |
| --- | --- | --- | --- |
| Generalized anxiety | 19 | 25 | 26 |
| Social anxiety | 13 | 17 | 17 |
| Depression | 10 | 13 | 12 |
| Separation anxiety | 10 | 10 | 11 |
| Specific phobia | 5 | 5 | 5 |
| Agoraphobia | 5 | 5 | 5 |
| PTSD | 4 | 3 | 4 |
| OCD | 3 | 3 | 3 |
| Panic disorder | 2 | 2 | 2 |

| **Number of anxiety diagnoses** | **Anticipation (n=70)** | **Negative feedback (n=78)** | **Positive feedback (n=81)** |
| --- | --- | --- | --- |
| 0 | 40 | 43 | 44 |
| 1 | 14 | 15 | 16 |
| 2 | 6 | 10 | 11 |
| 3 or more | 10 | 10 | 10 |

**Appendix 2. Analyses with SDQ emotional problems.**

**REWARD ANTICIPATION**

**Table S2A.** The effects of ASD traits and emotional problems on BOLD responses during reward anticipation. Confirmatory analyses with the SDQ emotional problems subscale showed similarities to the anxiety analyses presented in main text. Consistent with anxiety findings, we found an interaction between ASD traits and emotional problems in the right insula and middle temporal gyrus (MTG; with ASD_EMOT_ showing the largest activation). We also found that youth with high levels of emotional problems showed increased activation in the right middle frontal gyrus (MFG), consistent with the main effect of anxiety found above. The main effect of ASD in frontal regions, found in anxiety analyses, did not reach significance (*p_FWE_*=.144, *k*=109). However, the same areas were found in ASD_EMOT_ > EMOT_ONLY_ and ASD_ANX_ > ANX_ONLY_ comparisons (left insula, left IFG, bilateral cuneus). Only one cluster (right MTG) remained significant for comparison ASD_EMOT_ > ASD_ONLY_ relative to ASD_ANX_ > ASD_ONLY_. Additionally, ASD_EMOT_ displayed increased activity in right thalamus and caudate relative to ASD_ONLY_.

|  | |  | |  | | peak MNI coordinates | | | | | |  | |  | |
| --- | --- | --- | --- | --- | --- | --- | --- | --- | --- | --- | --- | --- | --- | --- | --- |
| **Region** | | **Brodmann Area** | | **Cluster size in voxels (*k*)** | | **x** | | **y** | | **z** | | **Z** | | ***p* (FWE)** | |
|  | | | | | |  | |  | |  | |  | |  | |
| **Interaction: ASD traits x Emotional problems** | | | | | | | | | | | | | | |  |
|  |  | |  | |  | |  | |  | |  | |  | |  |
| R thalamus, R caudate, R insula, R middle temporal gyrus | 13 | | 306 | | 15 | | -13 | | 10 | | 4.30 | | <.001 | |  |
|  |  | |  | | 51 | | -40 | | -5 | | 3.67 | |  | |  |
|  |  | |  | | 69 | | -25 | | -17 | | 3.61 | |  | |  |
|  | | | | | | | | | | |  | |  | | |
| R medial and superior frontal gyri/SMA, R midcingulate gyrus/dorsal ACC | 6/8/32 | | 237 | | 9 | | -1 | | 58 | | 3.54 | | .002 | |  |
|  |  | |  | | 0 | | 17 | | 52 | | 3.49 | |  | |  |
|  |  | |  | | 24 | | -16 | | 58 | | 3.33 | |  | |  |
|  | | | | | | | | | | |  | |  | | |
| **High > Low ASD traits** | | | | | | | | | | | |  | |  | |
|  | |  | |  | |  | |  | |  | |  | |  | |
| L insula extending to L inferior frontal gyrus | | 13/47 | | 249 | | -33 | | 35 | | 13 | | 4.07 | | .002 | |
|  |  |  | |  | | -36 | | 14 | | 1 | | 3.97 | |  | |
|  | |  | |  | | -42 | | 47 | | 1 | | 3.34 | |  | |
|  | |  | |  | |  | |  | |  | |  | |  | |
| L posterior cingulate gyrus, L cuneus | | 31/18 | | 252 | | -6 | | -67 | | 13 | | 3.77 | | .001 | |
|  | |  | |  | | -9 | | -82 | | 13 | | 3.65 | |  | |
|  | |  | |  | | -15 | | -79 | | 22 | | 3.43 | |  | |
|  | |  | |  | |  | |  | |  | |  | |  | |
| **High > Low emotional problems** | | | | | | | |  | |  | |  | |  | |
|  | |  | |  | |  | |  | |  | |  | |  | |
| R middle and inferior frontal gyri | | 47 | | 141 | | 45 | | 26 | | -14 | | 4.02 | | .047 | |
|  | |  | |  | | 39 | | 41 | | -11 | | 3.48 | |  | |
|  | |  | |  | | 51 | | -40 | | -5 | | 3.37 | |  | |
|  | |  | |  | |  | |  | |  | |  | |  | |
| **ASD_EMOT_ > ASD_ONLY_** | | | | | | | | | | | | | | | |
|  | |  | |  | |  | |  | |  | |  | |  | |
| R thalamus, R caudate | |  | | 138 | | 12 | | -13 | | 10 | | 3.64 | | .022 | |
|  | |  | |  | | 27 | | -31 | | 16 | | 3.47 | |  | |
|  | |  | |  | | 27 | | -22 | | 1 | | 3.31 | |  | |
|  | |  | |  | |  | |  | |  | |  | |  | |
| R middle temporal gyrus | | 21 | | 131 | | 54 | | -16 | | -17 | | 3.49 | | .030 | |
|  | |  | |  | | 60 | | -55 | | -2 | | 3.30 | |  | |
|  | |  | |  | | 72 | | -28 | | -17 | | 3.21 | |  | |
|  | |  | |  | |  | |  | |  | |  | |  | |
| **ASD_EMOT_ > EMOT_ONLY_** | | | | | | | | | | | | | | | |
|  | |  | |  | |  | |  | |  | |  | |  | |
| L insula extending to L inferior frontal gyrus | | 13/47 | | 201 | | -36 | | 17 | | 1 | | 4.47 | | .003 | |
|  |  |  | |  | | -42 | | 17 | | -5 | | 4.10 | |  | |
|  | |  | |  | | -45 | | 2 | | 1 | | 3.71 | |  | |
|  | |  | |  | |  | |  | |  | |  | |  | |
| L and R lingual gyrus, L and R cuneus extending to the posterior cingulate | | 18/17 | | 344 | | -6 | | -67 | | 13 | | 3.76 | | <.001 | |
|  |  |  | |  | | 6 | | -76 | | -5 | | 3.66 | |  | |
|  | |  | |  | | -27 | | -73 | | 4 | | 3.57 | |  | |
|  | |  | |  | |  | |  | |  | |  | |  | |

*ACC*, anterior cingulate cortex. *ASD_EMOT_*, high ASD traits & emotional problems. *ASD_ONLY_*, high ASD traits, low emotional problems. *EMOT_ONLY_*, high emotional problems & low ASD traits. *FWE*, family-wise error correction. *L*, left hemisphere. *R*, right hemisphere. *SMA*, supplementary motor area.

**NEGATIVE FEEDBACK**

**Table S2B.** The effects of ASD traits and emotional problems on BOLD responses patterns following negative feedback. Confirmatory analyses with SDQ emotional problems subscale brought similar results to our anxiety analyses (presented in main text). Consistent with anxiety findings, we found an interaction between ASD traits and emotional problems in right MFG and IFG. We also found the main effect of ASD traits in right medial and superior frontal gyri. Youth with high levels of emotional problems showed decreased activation in frontal regions (bilateral IFG, MFG, SFG), consistent with the main effect of anxiety. Some of the same areas were found in ASD_EMOT_ < ASD_ONLY_ and ASD_ANX_ < ASD_ONLY_ comparisons (bilateral IFG, right MFG, left precentral gyrus); and ASD_EMOT_ < TD_EMOT_ and ASD_ANX_ < TD_ANX_ (right caudate, right medial and superior frontal gyri).

|  |  |  | peak MNI coordinates | | |  |  |
| --- | --- | --- | --- | --- | --- | --- | --- |
| **Region** | **Brodmann Area** | **Cluster size (voxels)** | **x** | **y** | **z** | **Z** | ***p* (FWE)** |
|  | | |  |  |  |  |  |
| **Interaction: ASD traits x Emotional problems** | | | | | |  |  |
|  | | | | | |  |  |
| R inferior and middle frontal gyri, R dorsal anterior cingulate gyrus | 10/32 | 314 | 39 | 35 | 10 | 4.07 | .002 |
|  |  |  | 18 | 26 | 13 | 3.96 |  |
|  |  |  | 48 | 38 | 10 | 3.94 |  |
|  |  |  |  |  |  |  |  |
| **High < Low ASD traits** |  |  |  |  |  |  |  |
|  |  |  |  |  |  |  |  |
| R medial and superior frontal gyri | 10 | 200 | 21 | 44 | 1 | 4.50 | .024 |
|  |  |  | 18 | 53 | 10 | 4.34 |  |
|  |  |  | 30 | 56 | -2 | 4.23 |  |
|  |  |  |  |  |  |  |  |
| **High < Low emotional problems** | | | |  |  |  |  |
|  |  |  |  |  |  |  |  |
| L midcingulate gyrus, corpus callosum | 24 | 198 | -15 | 5 | 31 | 4.67 | .025 |
|  |  |  | -6 | -7 | 28 | 3.91 |  |
|  |  |  | -15 | -10 | 28 | 3.48 |  |
|  |  |  |  |  |  |  |  |
| L and R inferior, middle and superior frontal gyri (extending to dACC), R insula, R putamen, R precentral and superior temporal gyri | 9/10/46/45/44/13 | 2074 | 27 | 32 | 31 | 4.55 | <.001 |
|  |  |  | 63 | -25 | 19 | 4.51 |  |
|  |  |  | 21 | 53 | 1 | 4.49 |  |
|  |  |  |  |  |  |  |  |
| **ASD_EMOT_ < ASD_ONLY_** | | | | | | | |
|  |  |  |  |  |  |  |  |
| R inferior frontal and precentral gyri, sub-gyral (R frontal lobe) |  | 950 | 42 | 35 | 10 | 4.70 | <.001 |
|  |  |  | 30 | 38 | 19 | 4.35 |  |
|  |  |  | 42 | -16 | 37 | 4.03 |  |
|  |  |  |  |  |  |  |  |
| L paracentral lobule, corpus callosum, L and R supplementary motor area | 6 | 626 | 0 | -19 | 70 | 4.17 | <.001 |
|  |  |  | -15 | 5 | 28 | 3.91 |  |
|  |  |  | -3 | -4 | 64 | 3.68 |  |
|  |  |  |  |  |  |  |  |
| L inferior and middle frontal gyri | 10/46 | 295 | -48 | 50 | 1 | 3.95 | <.001 |
|  |  |  | -45 | 44 | 25 | 3.49 |  |
|  |  |  | -27 | 68 | 25 | 3.44 |  |
|  |  |  |  |  |  |  |  |
| L precentral, inferior frontal, and superior temporal gyri | 22/9 | 201 | -60 | 2 | 4 | 4.17 | .007 |
|  |  |  | -60 | 8 | 28 | 3.71 |  |
|  |  |  | -48 | 2 | 28 | 3.61 |  |
|  |  |  |  |  |  |  |  |
| **ASD_EMOT_ < EMOT_ONLY_** |  |  |  |  |  |  |  |
|  |  |  |  |  |  |  |  |
| R caudate, R medial and superior frontal gyri extending to the anterior cingulate | 10 | 317 | 15 | 59 | 7 | 4.75 | <.001 |
|  |  |  | 27 | 59 | -2 | 4.26 |  |
|  |  |  | 15 | 50 | 1 | 3.86 |  |
|  |  |  |  |  |  |  |  |

*ASD_EMOT_*, high ASD traits & emotional problems. *ASD_ONLY_*, high ASD traits, low emotional problems. *EMOT_ONLY_*, high emotional problems & low ASD traits. *dACC*, dorsal anterior cingulate cortex. *FWE*, family-wise error correction. *L*, left hemisphere. *R*, right hemisphere.

**POSITIVE FEEDBACK**

**Table S2C.** The effects of ASD traits and emotional problems on brain activation patterns following positive feedback.

|  |  |  | peak MNI coordinates | | |  |  |
| --- | --- | --- | --- | --- | --- | --- | --- |
| **Region** | **Brodmann Area** | **Cluster size (voxels)** | **x** | **y** | **z** | **Z** | ***p* (FWE)** |
|  | | |  |  |  |  |  |
| **High < Low emotional problems** | | | | | |  |  |
|  |  |  |  |  |  |  |  |
| L middle occipital gyrus |  | 191 | -51 | -70 | -8 | 4.10 | .030 |
|  |  |  | -48 | -82 | 7 | 3.68 |  |
|  |  |  | -30 | -70 | 10 | 3.41 |  |
|  |  |  |  |  |  |  |  |

*FWE*, family-wise error correction. *L*, left hemisphere.

**Appendix 3. Longitudinal predictions.**

**REWARD ANTICIPATION**

**Table S3A.** Predicting anxiety status (no/yes) at two-year follow-up by right medial frontal gyrus (medFG) and right Brodmann area 32 (BA32) activations during reward anticipation at baseline. Presented separately for participants with high and low ASD traits.

|  |  | **ROI: right medFG** | | **ROI: right BA 32** | |
| --- | --- | --- | --- | --- | --- |
| Variable |  | *Odds Ratio (95% Confidence Interval)* | | | |
|  |  | High ASD traits | Low ASD traits | High ASD traits | Low ASD traits |
|  |  |  |  |  |  |
| ROI |  | 62.33* (1.46-2668.54) | 0.93 (0.63-1.39) | 33.22* (1.47-750.36) | 0.84 (0.55-1.28) |
| female gender |  | 12.10* (1.37-107.13) | 1.84** (1.29-2.61) | 7.81* (1.07-56.91) | 1.83** (1.28-2.60) |
| handedness |  | 1.06 (0.13-8.82) | 1.49 (0.86-2.58) | 0.96 (0.13-7.34) | 1.48 (0.85-2.57) |
| site |  | 1.09 (0.76-1.58) | 1.03 (0.96-1.11) | 1.10 (0.78-1.55) | 1.03 (0.96-1.11) |
| WISC Verbal Comprehension |  | 1.09 (1.00-1.18) | 1.00 (0.98-1.01) | 1.07 (1.00-1.15) | 1.00 (0.98-1.01) |
| WISC Reasoning |  | 1.00 (0.94-1.06) | 0.99 (0.98-1.00) | 1.01 (0.95-1.07) | 0.99 (0.98-1.00) |
| Baseline ASD traits |  | 1.13 (0.96-1.33) | 1.02 (0.93-1.12) | 1.13 (0.96-1.33) | 1.02 (0.93-1.12) |
| Baseline anxiety (“yes”) |  | 16.29* (1.70-155.60) | 5.50*** (3.89-7.78) | 12.15* (1.51-97.98) | 5.49*** (3.88-7.76) |
|  |  |  |  |  |  |
| LR Χ^2^ |  | 23.36** | 121.97*** | 23.04** | 122.49*** |
| Pseudo R^2^ |  | 0.36 | 0.12 | 0.36 | 0.12 |
| Log likelihood |  | -20.37 | -437.71 | -20.53 | -437.45 |

*p < .05; **p < .01; ***p<.001. *ROI*, region-of-interest.

**References**

1. Knutson B, Westdorp A, Kaiser E, Hommer D (2000): FMRI visualization of brain activity during a monetary incentive delay task. *Neuroimage*. 12:20-27.

2. Schumann G, Loth E, Banaschewski T, Barbot A, Barker G, Büchel C, et al. (2010): The IMAGEN study: reinforcement-related behaviour in normal brain function and psychopathology. *Mol Psychiatry*. 15:1128-1139.
